# Raman Spectroscopic Tools to Probe the Skin–(Trans)dermal Formulation Interface

**DOI:** 10.1021/acs.molpharmaceut.2c00480

**Published:** 2022-09-06

**Authors:** Hazel Garvie-Cook, Magdalena Hoppel, Richard H. Guy

**Affiliations:** Department of Life Sciences, University of Bath, Claverton Down, Bath BA2 7AY, U.K.

**Keywords:** Raman spectroscopy, Raman imaging, topical skin formulations, transdermal drug delivery, skin bioavailability

## Abstract

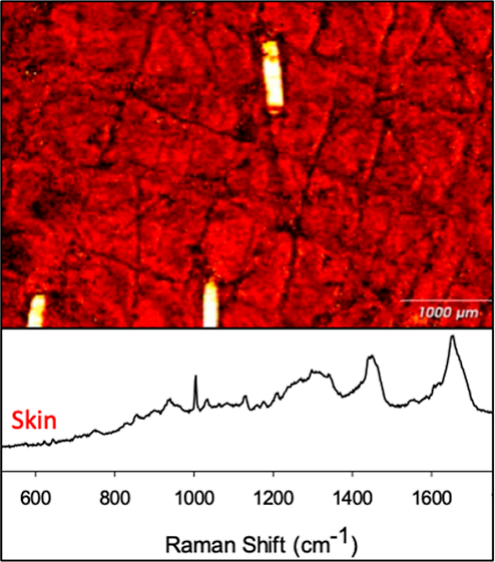

Medicines designed to deliver the active pharmaceutical ingredient either into or through the skin—often referred to as topicals and transdermals, respectively—are generally considered to be complex drug products. A particular challenge faced by these formulations is identifying a suitable method (ideally, in terms of specificity, accuracy, precision, and robustness) or combination of methods with which to assess the amount and rate of drug delivery to the target site. Significant research currently aims to identify and validate relevant and minimally invasive techniques that can be used to quantify both the levels of the drug attained within different parts of the skin and the kinetics with which the drug is taken up into the skin and cleared therefrom into the systemic circulation. Here, the application of confocal Raman microspectroscopy and imaging to interrogate events integral to the performance of topical and transdermal drug products at the formulation-skin interface is illustrated. Visualization, depth slicing, and profiling are used (a) to elucidate key chemical properties of both the delivery system and the skin that have impact on their interaction and the manner in which drug transfer from one to the other may occur, (b) for the transformation of a drug product from that manufactured into a residual phase post-application and inunction into the skin (including the potential for important changes in solubility of the active compound), and (c) for drug absorption into the skin and its subsequent ‘“clearance” into deeper layers and beyond. Overall, the Raman tools described offer both qualitative and potentially semi-quantitative insights into topical and transdermal drug product performance and provide information useful for formulation improvement and optimization.

## Introduction

To optimize the delivery of pharmacologically active compounds into and across the skin, a suitable analytical method is required to probe both the interactions occurring at the interface between the skin and the (trans)dermal formulation and the subsequent percutaneous transport of the drug. Of specific interest is the ability to determine the rate and extent of drug absorption at or close to its site of action (i.e., the bioavailability) either within a particular part of the skin, for example, the viable epidermis or in the subcutaneous tissue or systemically.^1^ The latter is the domain of subcutaneous and transdermal administration involving the application of a patch (or more typical semisolid formulations) to deliver the drug at a controlled rate and elicit a central pharmacological effect. In this case, the transfer of the drug from patch to skin and its subsequent diffusion through the outer stratum corneum (SC) layer are the factors which determine bioavailability.^2^ Conventional topical products (gels, creams, ointments, etc.) which are designed to deliver the drug to treat local skin conditions undergo significant changes as they are rubbed onto the skin,^3^ in contrast to “static” transdermal patches. In other words, the vehicle changes dramatically from that in the finished product applied to a residual film left on the skin surface, once volatile (and other) excipients have either been lost by evaporation and/or uptake into the SC.^4^ It is now well recognized that this metamorphosis of a formulation has a significant impact on drug uptake and permeation into and through the skin.^5^

There is significant impetus, therefore, to identify tools with which events at the drug delivery system–skin interface can be studied, and Raman spectroscopy, in particular, has emerged as a non-destructive and label-free method with considerable potential for exploitation in this area.^6^ Indeed, major advances in spectral acquisition and data analysis have enabled Raman to better probe specific chemical species present in complex environments,^7,8^ to achieve at least semi-quantitative characterization of target analytes^9^ and to visualize their spatial and temporal disposition in three dimensions.^10,11^

The research described in this paper first applies Raman spectroscopy and imaging to examine the components of an approved transdermal nicotine patch and the upper layers of the skin and then to characterize the impact of applying the delivery system and the release of the drug into the stratum corneum. The potential of the tool to more clearly understand and visualize the transformation of two example topical formulations when applied to the skin is then demonstrated; one is a topical film-forming system containing betamethasone valerate, and the other is a marketed acyclovir cream. In this way, the study provides further support for using Raman spectroscopy to investigate formulation–skin interactions at the interface and validates the future application of this spectroscopic tool to better understand the complexities involved in achieving efficient drug delivery to and across the skin.

## Materials & Methods

### Materials

Porcine skin, which is a recognized and representative model for the human counterpart,^13^ was used in all experiments. Dorsal skin from a single pig was obtained from a local slaughterhouse shortly after sacrifice of the animal and was subsequently cleaned in cold water and dermatomed (Zimmer, Warsaw, IN) to a thickness of approximately 750 μm. The skin sections were then individually wrapped in Parafilm and placed in a freezer at −20 °C. Shortly before use, the skin was thawed, and excess hair was carefully clipped using scissors. Nicotinell Step 2 patches (GlaxoSmithKline Consumer Healthcare, UK) were acquired from Boots UK Limited (Nottingham, UK). Zovirax (which contains 5% w/w acyclovir) was from GlaxoSmithKline Consumer Healthcare (Brentford, UK). Betamethasone-17-valerate (BMV, purity 100%) was purchased from Crystal Pharma SAU (Boecillo, Spain). Eudragit RS PO (Eudragit) (ammonio methacrylate copolymer type B) was supplied by Evonik Röhm GmbH (Darmstadt, Germany), Klucel LF (Klucel) (hydroxypropyl cellulose) by Azelis (Lyngby, Denmark), medium-chain triglyceride (Miglyol 812 N, caprylic/capric triglyceride) by Sasol (Hamburg, Germany), and triethyl citrate (TEC) by Merck (Darmstadt, Germany).^12^

### Raman Spectroscopy

#### Imaging

Raman images of a nicotine patch prior to application and of porcine skin after the removal of a nicotine patch were acquired. Imaging was performed using the RA802 Pharmaceutical Analyser (Renishaw, Gloucestershire, UK) which uses an excitation wavelength of 785 nm and a focused laser line to acquire a grid of Raman spectra over the surface of the sample. Focus-tracking (LiveTrack) was used to maintain focus on the surface of the sample, and data was acquired with a 50× long working distance objective.

To prepare the nicotine patch for imaging, the liner was removed, and the patch was placed on a stainless-steel slide with the adhesive layer facing up. Imaging (StreamLine) was performed over an area of ∼7.5 mm × 5.0 mm, using a step size (pixel size) of 20 μm. The total number of spectra in the imaging set was ∼93,000.

Analysis of the nicotine patch imaging data was performed using WiRE software (Renishaw) and Empty Modelling component analysis, an unsupervised multivariate curve resolution-alternating least squares method (MCR-ALS), to extract identifiable component spectra from Raman datasets. Components were identified in WiRE by comparing them with spectra of known materials in spectral libraries. Images were then generated using direct classical least squares (DCLS) component analysis and the reference spectra for the identified components.

**Figure 1:**
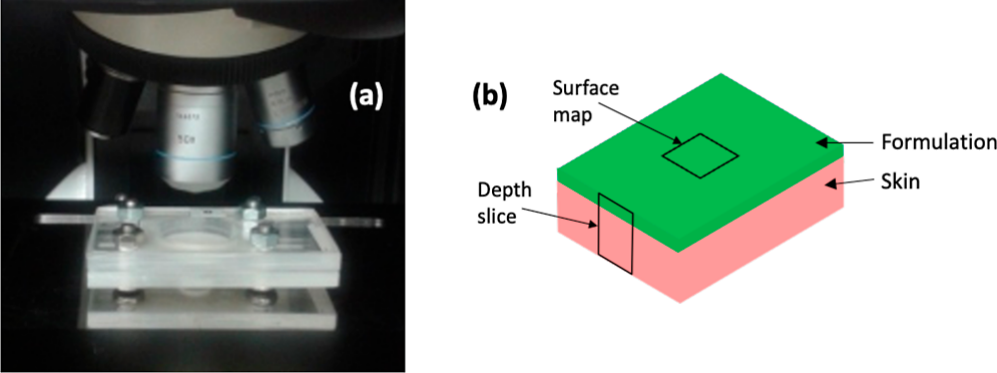
(a) Purpose-made holder to enable the lower surface of the skin to be bathed with a physiological buffer, while acquiring Raman spectra. The hole cut in the top of the holder was sufficiently large to permit the objective to approach close to the surface of the skin. (b) Schematic diagram illustrating the depth slice and surface mapping measurements.

To prepare the skin for imaging, a nicotine patch was applied for 2 h to a tissue sample positioned over a physiologically relevant phosphate-buffered saline bath at pH 7.4 in a custom-built holder (Figure 1). At the termination of the treatment, the patch was removed, the skin was placed on a stainless-steel slide, and focus-tracking was again used to maintain focus over the uneven surface of the skin. Depth profiles were obtained immediately and 1.5 and 3 h later. Imaging (StreamLine) was performed over an area of ∼7.0 mm × 4.8 mm, with a step size of 20 μm. The total number of spectra in the imaging set was ∼84,600. All measurements were acquired in triplicate under ambient laboratory conditions (temperature ∼21 °C, relative humidity ∼45%). While skin integrity was visually inspected for defects, no instrumental assessment of barrier function (e.g., transepidermal water loss or skin resistance) was made.

Analysis of the skin imaging data was performed in WiRE using principal component analysis (PCA), a multivariate analysis method to distinguish spectral trends in the data and to generate images showing changes in the spectra over the analyzed area. Images show PCA scores, with positive scores representing spectra that more closely resemble the positive features of the PCA loading. Univariate analysis (Intensity at a Point) was also conducted to generate images based on spectral intensities.

### Depth Slices

Depth slices were acquired using a Raman microscope (inVia Qontor, Renishaw, Gloucestershire, UK) to determine the distribution of formulations on the surface of the skin. For Raman measurements, acyclovir and betamethasone valerate (BMV) formulations were applied to skin in the same setup used for the nicotine patch (Figure 1).

Depth slices, with a step size of 1 μm, were acquired after a 4 h application of the acyclovir cream and of the BMV film-forming systems. For the former, the Zovirax product was applied uniformly to the skin surface as “a thin layer”, in accordance with the Patient Information Leaflet (https://www.medicines.org.uk/emc/product/5468/smpc). The preparation of the latter and their administration have been fully described in the literature.^14,15^ Measurements were performed in high confocal mode using a 50× objective, with an excitation wavelength of 633 nm.

Analysis of the skin imaging data was performed in WiRE using direct classical least squares (DCLS) component analysis, in combination with previously acquired reference spectra for the skin, drug, and key formulation components. This analysis method generates scores which reflect the contribution of each reference spectrum to the spectrum under analysis. The procedure was performed with normalization; that is, absolute intensities were not considered, and the generated images showed the presence, rather than the concentration, of the active ingredient. A second order background polynomial was included in the analysis.

### Depth Profiles

Depth profiles were acquired using a Raman microscope (inVia Qontor, Renishaw, Gloucestershire, UK) to determine the distribution of nicotine as a function of depth into the skin. A nicotine patch was applied to the skin for 2 h and then removed, and depth profiles were obtained immediately and 1.5 and 3 h later. Measurements were performed in high confocal mode using a 100× objective with an excitation wavelength of 633 nm. Spectra were acquired at depths of 0, 4, 8, and 12 μm below the skin. The “0” depth is the skin surface and was set when the microscope was directly focused here. The total time for each depth profile was approximately 12 min, with an exposure time of 180 s per spectrum. Three replicate depth profiles were acquired per time point.

Depth profile data (consisting of a spectrum for each depth) were analyzed in WiRE software (Renishaw, Gloucestershire, UK) using DCLS component analysis, in combination with previously acquired reference spectra for nicotine and skin. Analysis was performed without normalization, meaning absolute intensities were considered, and profiles dependent on the concentration were obtained. A second order background polynomial was again included in the analysis.

To account for signal loss with depth, DCLS analysis with the skin reference spectrum was used to analyze a “control” depth profile of untreated skin acquired in the same way. The DCLS component analysis score of skin decreased with depth as the signal originating from increasingly deeper regions of the skin was progressively attenuated. This approach allowed a normalization factor that maintained the skin signal at a fixed level to be determined. This factor was then applied to correct the DCLS scores of nicotine in the skin. This control measurement was also used to determine a limit of detection for nicotine; the DCLS score for nicotine determined when there was no nicotine in the skin (the control measurement) was considered the limit of detection (any scores lower than this were therefore considered to be reflective of no nicotine).

## Results & Discussion

### Imaging

#### Nicotine Patch

**Figure 2:**
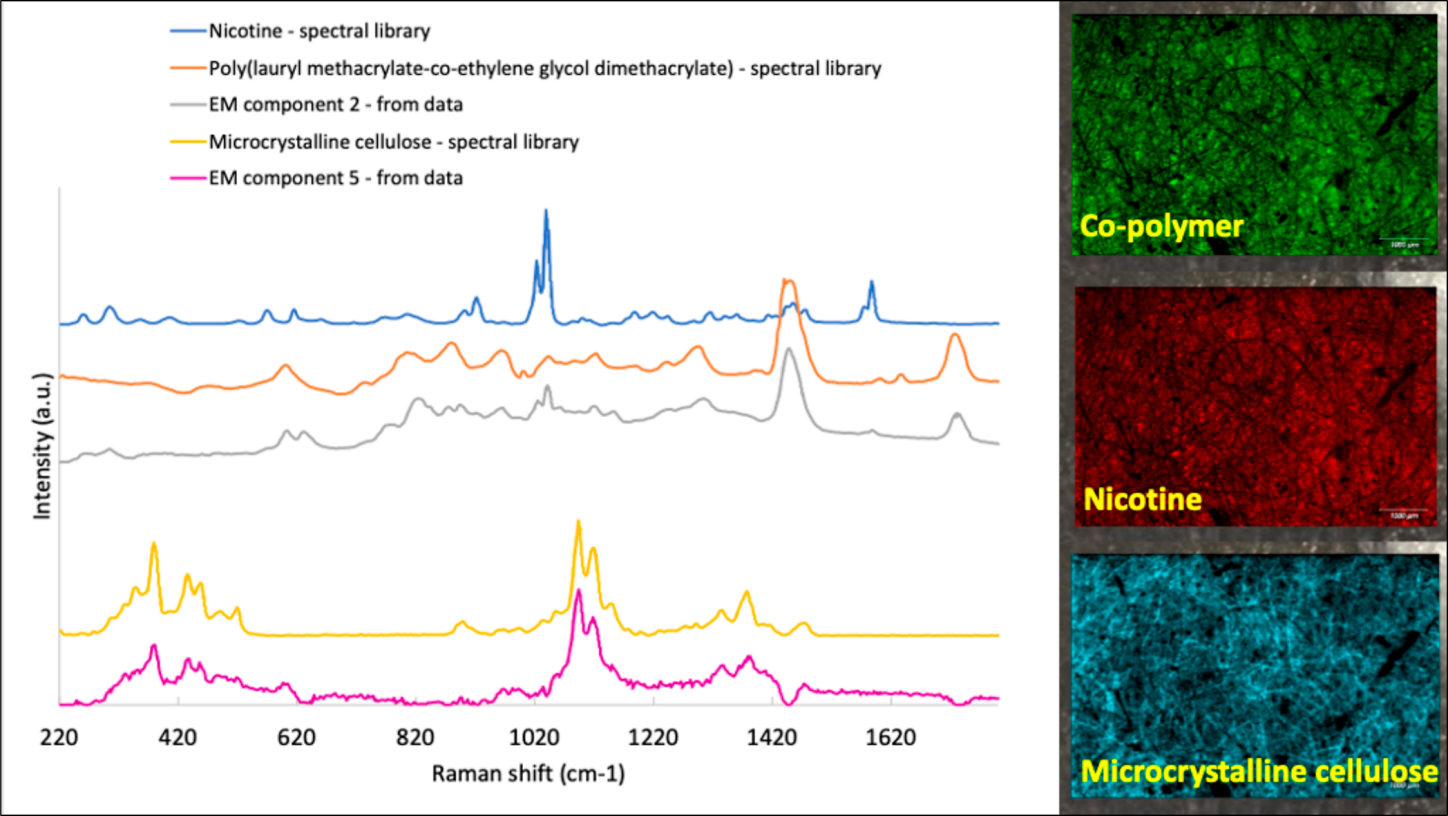
Empty Modelling (EM) component analysis revealed components in the nicotine patch that were identified as an acrylate co-polymer, the drug, and microcrystalline cellulose. Images on the right, acquired from an unused patch, revealed the distribution of these constituents.

Empty Modelling component analysis distinguished components that were identified as reflective of an acrylate co-polymer (although almost certainly not an exact match for that in the patch), nicotine, and microcrystalline cellulose (Figure 2).

Images generated for these components showed that these three key components are relatively evenly distributed in the patch, prior to its utilization (Figure 2). Typically, patches are formulated to achieve a drug concentration in the polymer matrix that is close to its solubility limit.^2^ This ensures that the drug flux delivered across the skin is sufficient to achieve a therapeutically effective delivery rate for a sustained period of time. The microcrystalline cellulose in the patch is the essential component of the “pad”, listed in the summary of the product’s characteristics (https://www.medicines.org.uk/emc/product/389/smpc).

Clearly, the combination of spectroscopy and imaging demonstrated here can also be used to characterize the distribution and subsequent release of other excipients that might be incorporated in a patch either to enhance drug solubility in or to increase drug diffusivity through the stratum corneum. Notably, for nicotine, the molecule is really quite soluble in the skin barrier layer and diffuses through it faster than any other currently approved transdermal drug,^2^ such that no enhancers are required.

### Skin after Removal of the Patch

**Figure 3:**
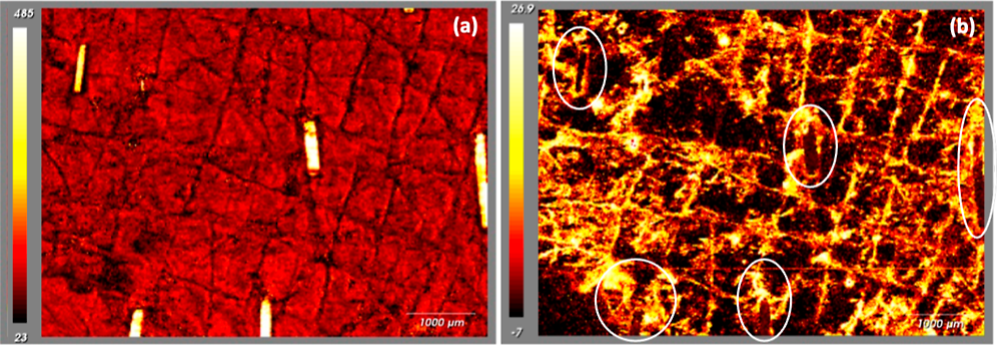
(a) Raman image of the amide I (i.e., keratin) signal intensity at 1659 cm^–1^ across the analyzed area; on the color scale, white represents a high concentration of the protein, with the clipped hair very clearly demarcated, while black indicates that a low concentration of keratin is apparent in the skin furrows. (b) PCA of the Raman image of skin shown in (a). Components are identified for lipids (high and positive PCA scores corresponding to yellow–white colors) and for protein (negative PCA scores and dark colors). Lipid is therefore seen fairly generally across the skin surface, with a noticeable concentration in the skin furrows and at the opening of hair follicles; keratin is associated with exposed corneocytes and with the highlighted hair.

A Raman image of the skin after removal of the patch was first generated using the signal intensity of the amide I band at 1659 cm^–1^, which broadly represents the protein (and primarily keratin, of course) content across the mapped area (Figure 3a). The clipped hair showed the highest intensity (yellow/white) protein signal, with the next, ubiquitous (red) signal coming from the corneocytes of the stratum corneum. The lowest signal was observed in the (dark) skin furrows.

Principal component analysis distinguished regions of higher lipid content, relative to that of the protein. Notably, an image of this lipid component (Figure 3b, which is derived from exactly the same skin area as that in Figure 3a) shows increased levels in the skin furrows and around the base of the highlighted hair emerging from their follicular openings. It is reasonable to suggest that the intensity and locations of this important lipid signal arise from sebum, which originates in the follicle-associated sebaceous glands and is excreted, via the follicles, onto the skin surface and subsequently accumulates in the furrows. Observations such as these are relevant with respect to drug delivery in which the first point of contact of an applied formulation will occur with the layer of sebum on the surface of the skin, and the initial “interfacial” transfer of drug from the vehicle will be sensitive to its affinity to this very lipophilic environment.

It was not possible to image nicotine at the skin surface in this way as the Raman signal strength was too weak; longer exposure times or the use of higher nicotine loadings may enable such information to be acquired.

### Depth Slices

**Figure 4:**
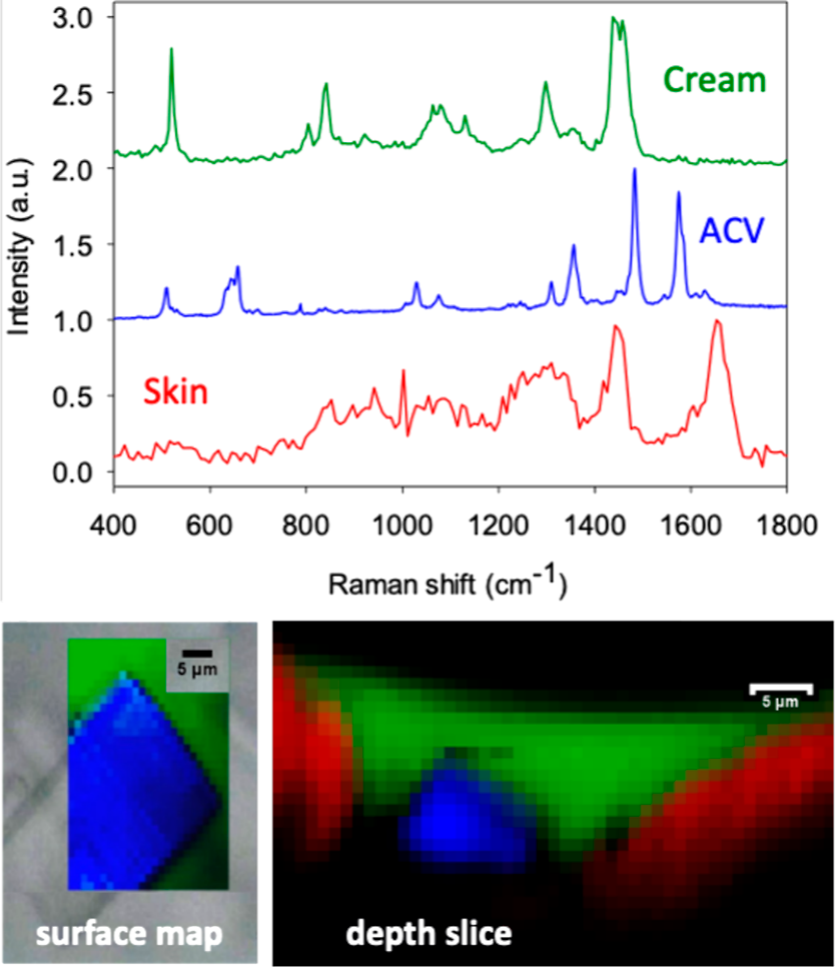
Reference Raman spectra of the acyclovir (ACV) cream, the pure, solid drug, and the skin. The left surface map image visualizes a clearly outlined ACV particle identified unequivocally by its Raman spectral signature (in blue) and superposed over that from the cream (in green). The right depth profile shows the cream-skin (in red) interface in the region of a skin furrow and again reveals the presence of a micron-sized ACV particle.

A surface map and depth slice were first obtained following application of acyclovir (ACV) cream (containing 5% w/w of the drug) to porcine skin ex vivo. To analyze the resulting data, reference spectra of the formulation, pure drug, and skin were acquired (Figure 4). As observed in the left image in Figure 4, the surface mapping image revealed the very obvious presence of solid ACV (coloured blue) on the skin surface post-administration. Such particles of drug themselves are clearly incapable of ever penetrating the stratum corneum. Even under a light microscope, the suspension nature of the cream (colored green) in the tube, in which it is supplied, is visually apparent (see background on the surface map in Figure 4),^1^ consistent with a recent report in the literature.^7^ The depth slice in the right image of Figure 4 illustrates the formulation-skin interface in the region of a skin furrow; the skin is colored in red. It is noted that the base of the furrow is too deep to be visible, the Raman sensitivity being insufficient to detect signals emanating from this point (and indicated by the pixels becoming black). Once again, however, a solid ACV particle of micron-sized dimensions is visible. This not surprising for this formulation, the delivery efficiency of which has been reported to be very low.^16,17^ Although this may be an extreme situation, it is possible to see how the application of Raman spectroscopy here could have a positive impact on the optimization of formulation design and that of the residual phase left on the skin post-application to ensure that delivery of the active compound can continue after metamorphosis of the vehicle during its rubbing onto the skin.^5^

**Figure 5:**
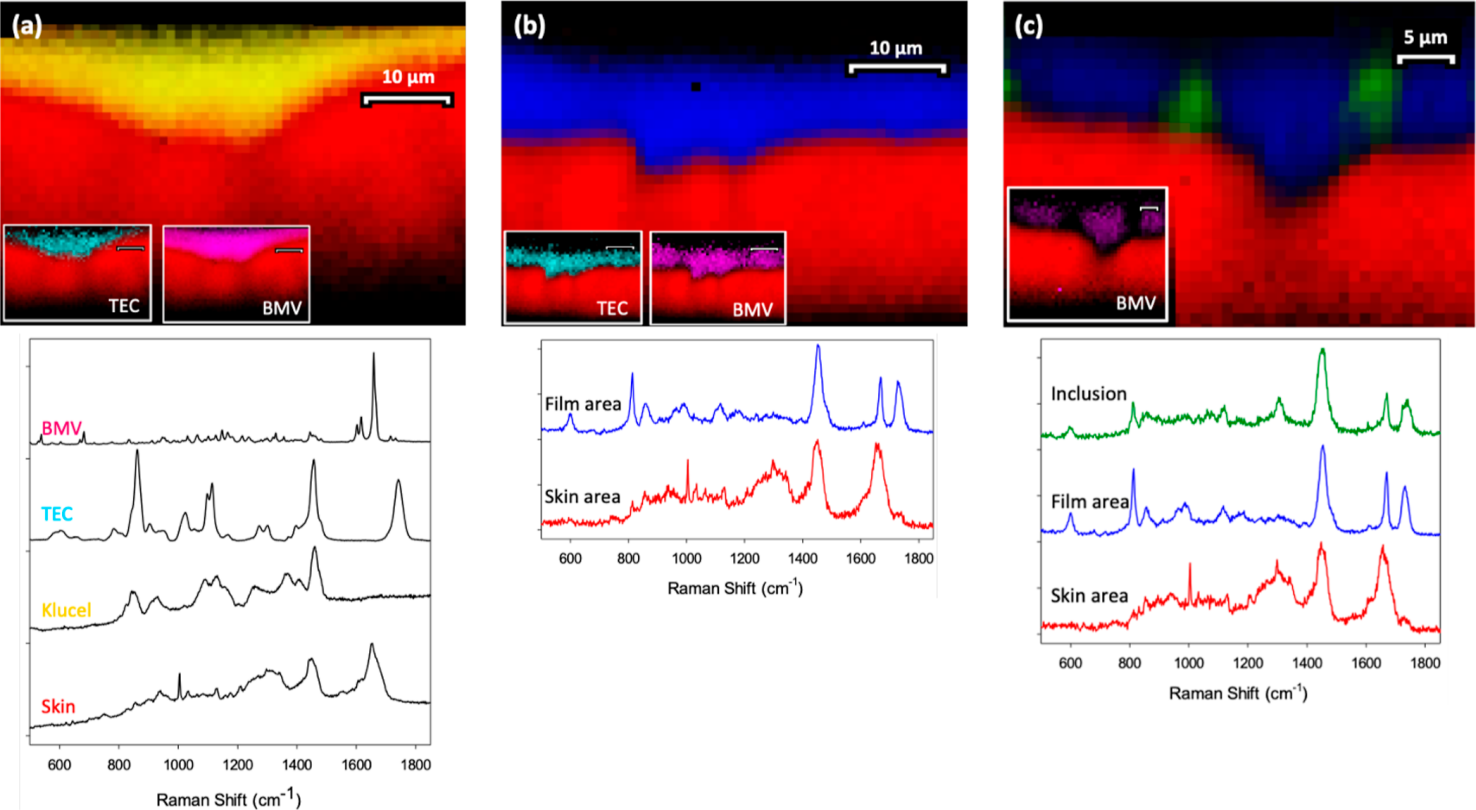
Reference spectra and representative depth slice images across the interface between skin (in red) and film-forming systems comprising (a) Klucel (yellow), TEC (turquoise), and BMV (magenta); (b) Eudragit (“Film area”, blue), TEC, and BMV; and (c) Eudragit, MCT (Inclusion, green) and BMV.

Depth slices were then acquired following the application of three film-forming systems containing the corticosteroid BMV. These formulations comprise, in addition to the drug, a polymer and a plasticizer. Films were formed after deposition of the ingredients in a volatile solvent (either ethanol or ethanol/water in a ratio of at least 15:1), the subsequent evaporation of which created the residual drug delivery system.^14,15^ Representative, Raman microscopic-derived depth slices are presented in Figure 5, together with the reference Raman spectra of the formulation components employed and the skin.

Figure 5a shows the residual formulation-skin interface for a film based on Klucel (shown in yellow), a relatively hydrophilic polymer containing the plasticizer, TEC. Post evaporation of the volatile solvent, the polymer conforms closely to the surface of the skin (again shown in red). The two inserts on the image show that both TEC (turquoise) and the drug (magenta) are well mixed into and uniformly distributed in the polymer. It is important to note that both the skin and BMV show significant Raman signals around 1700 cm^–1^, and it is appropriate to ask how this potential overlap is managed. The answer lies in the DCLS component analysis used to interpret these images; DCLS is a multivariate analysis method wherein multiple Raman peaks from both the skin and BMV are employed. This allows a very clear differentiation between the drug and the tissue because when viewed across the entire spectral range, the Raman profiles can be distinguished and separated from one another.

In Figure 5b, the polymer has been replaced by the more hydrophobic acrylate, Eudragit (the “Film area” shown in blue), which also conformed well to the skin. Furthermore, the distributions of TEC and BMV were again quite homogeneous, as shown in the insets. The formulation in Figure 5c was also based on Eudragit, but, in this case, the plasticizer used was changed to the more lipophilic MCT (the “Inclusion” shown in green). While the skin-polymer contact remained good, the distributions of MCT and BMV in the residual film were no longer uniform. There were clearly MCT-enriched inclusions in the film, as previously described in the literature based on nano-indentation and elastic modulus measurements made using atomic force microscopy and Raman chemical maps of the films deposited on glass slides.^14,15^ In contrast, as indicated in the inset, BMV was preferentially distributed in areas where less MCT was present. Once more, then, valuable information is accessed by application of the Raman imaging and characterization tools presented in this work. From the earlier in vitro (i.e., not involving skin) work,^14,15^ it is apparent that an inhomogeneous distribution of excipients can lead to differential drug levels and solubilities in different parts of the film and that this may ultimately have impact on drug release/delivery.

### Depth Profiles

**Figure 6:**
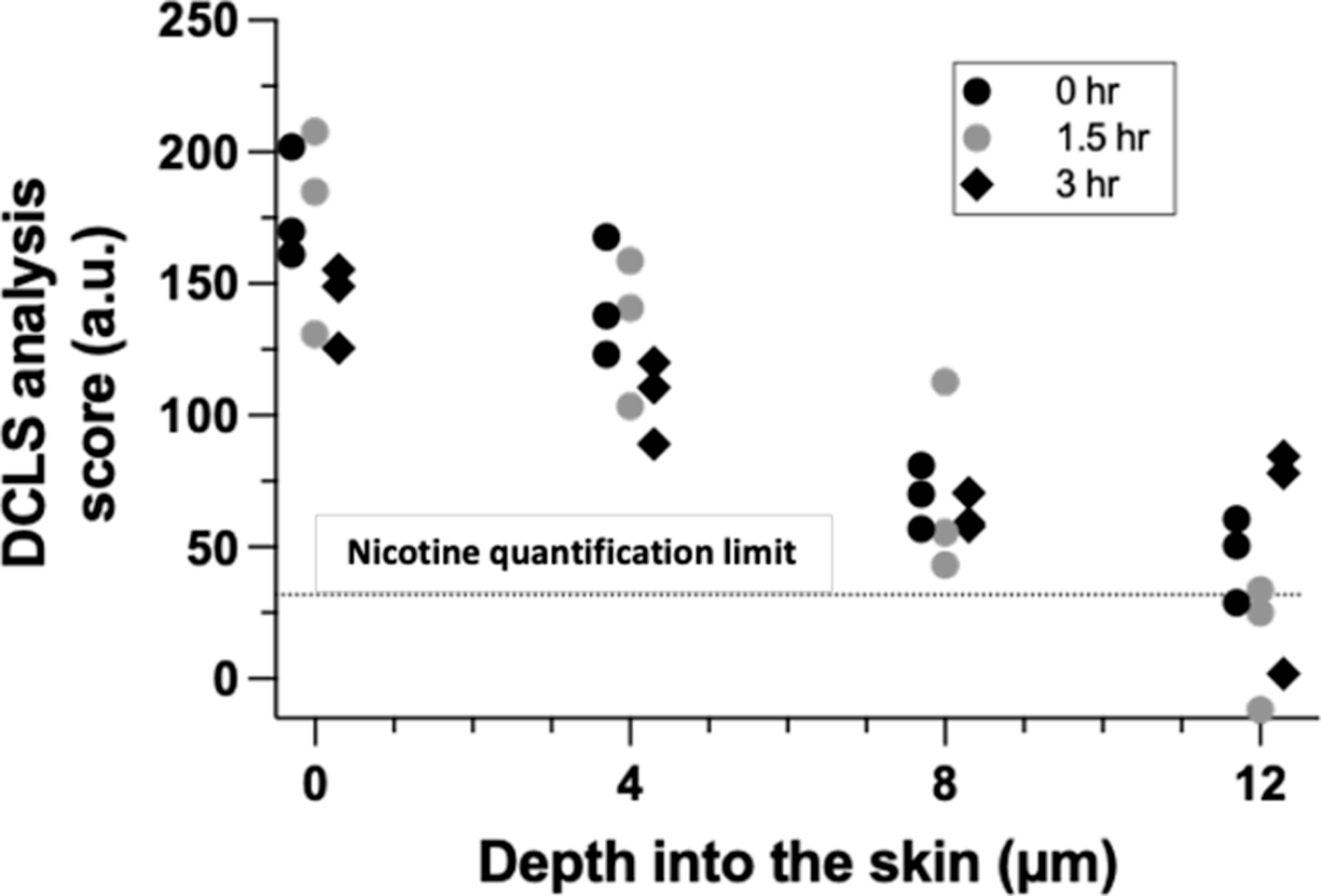
Nicotine “concentration” (expressed in terms of the measured DCLS analysis score) as a function of the position in the skin determined from Raman spectroscopy depth profiles immediately post-removal of the patch (0 h) and 1.5 and 3 h later. Depth profiles were acquired at three positions, designated by the different symbols used, at each time point.

Following a 2 h application of the nicotine patch, depth profiles of the drug across the stratum corneum were assessed immediately and 1.5 and 3 h later. The DCLS analysis score for the drug was assessed in triplicate each time at the skin surface and at three depths (4, 8, and 12 μm) into the tissue. The results are shown in Figure 6, which also highlight the DCLS limiting score at which reliable quantification of nicotine is no longer possible.

The drug profile across the stratum corneum upon removal of the patch appears to be close to linear, suggesting that nicotine transport was close to steady-state within the 2 h application period. This is consistent with the known, rapid permeation of nicotine across the skin.^2^ Subsequently, the “clearance” of the drug from the region of skin observed can be assessed from the attenuation of the sum of the measured DCLS analysis scores (over the four positions examined) as a function of time. When the natural logarithm of these cumulative scores is plotted against time (see Supporting Information), the slope of the resulting straight line can be used to deduce a first-order rate constant of 0.06 (±0.01) h^–1^, for nicotine elimination from the skin. While this value is smaller (and has been deduced from a relatively small number of replicates on skin from a single pig) than that recently reported in the in vivo stratum corneum sampling study in human volunteers using the tape-stripping methodology [0.37 (±0.17) h^–1^],^18^ it is within an order of magnitude. The absence of a functioning microcirculation in ex vivo porcine skin or the ability to react physiologically to the applied product (e.g., local irritation/vasodilatation) are potential explanations for the difference observed. Of general significance, though, is that the depth profiling can be used to provide metrics via the DCLS analysis score relevant to drug uptake and elimination from the skin, following the application of a topical formulation.

## Conclusions

The research described offers an illustrative overview of Raman spectroscopic and imaging tools that can probe and characterize events taking place at the interface between the skin and a topical or transdermal drug delivery system. Such information, which can be acquired rapidly and minimally invasively, is able to shed light on the chemical distribution and spatial localization of skin components/features and changes in formulation properties that can affect drug uptake into and penetration through the skin. While several research directions are implied by the observations presented here, a particular focus of future work is to examine the ability of Raman spectroscopy to compare the skin bioavailability of a drug when delivered from different formulations so as to assess their bio(in)equivalence.

## Supporting Information Available

Graph illustrating the “clearance” kinetics of nicotine from the skin, as assessed by confocal Raman spectroscopy (PDF)

## Footnotes

1 It is noteworthy, however, that the acyclovir signal from areas of the cream that were devoid of crystals was below the limit of quantification.
