# Supplementary material for: Raman Spectroscopic Tools to Probe the Skin–(Trans)dermal Formulation Interface
Source: Mol Pharm. 2022 Sep 6;19(11):4010–6. doi: 10.1021/acs.molpharmaceut.2c00480 (PMC9644382; doi:10.1021/acs.molpharmaceut.2c00480)
Supplement: Supplementary file 1 — mp2c00480_si_001.pdf [file mp2c00480_si_001.pdf]

SUPPORTING INFORMATION

Raman spectroscopic tools to probe the skin-(trans)dermal formulation interface

Hazel Garvie-Cook, Magdalena Hoppel and Richard H. Guy*

University of Bath, Department of Pharmacy & Pharmacology, Claverton Down, Bath, BA2 7AY, U.K.

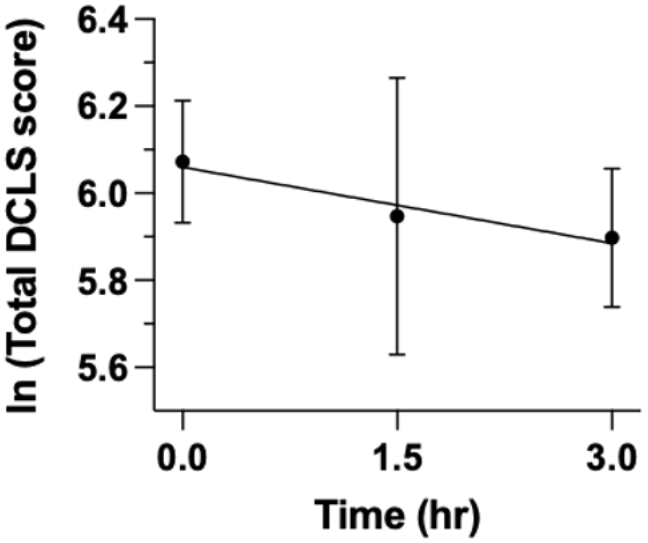

'Clearance' of nicotine from the skin interrogated by confocal Raman spectroscopy is determined from the slope of the natural logarithm of the attenuation of the sum of the measured DCLS analysis scores (over the four positions examined) as a function of time.
